# Magnetic resonance spectroscopy guided radiotherapy boost for patients with glioblastoma

**DOI:** 10.1038/s41598-025-87632-1

**Published:** 2025-04-18

**Authors:** Peng Xu, Dongmei Liu, Hongyuan Hu, Tong Liu, Chunmei Liang, Zhipeng Wen, Ke Yuan, Jun Yin, Lucia Clara Orlandini, Jinyi Lang

**Affiliations:** 1 School of Medicine, University of Electronic Science and Technology of China, Chengdu, China; 2 Department of Radiation Oncology, Sichuan Cancer Hospital & Institute, Sichuan Cancer Center, Affiliated Cancer Hospital of University of Electronic Science and Technology of China, Chengdu, China

**Keywords:** Glioblastoma, Boost, Magnetic resonance spectroscopy, MRS, Radiotherapy, Cancer, Oncology

## Abstract

To assess the feasibility and efficacy of delivering a radiation dose boost to high-risk glioblastoma (GBM) tumor regions, identified using magnetic resonance spectroscopy (MRS) after standard radiotherapy (RT). This retrospective study included patients newly diagnosed with GBM between January 2017 and July 2023. All patients received Intensity-modulated radiotherapy and concurrent temozolomide. Magnetic resonance imaging (MRI) and MRS were performed prior to radiotherapy and after the administration of 60Gy. A biological gross tumor volume (bGTV) was delineated on the simulation CT using the image fusion with the T1 sequence with MRS data after 60Gy. Based on the bGTV volume, patients received an additional 10-20Gy. Survival outcomes were estimated using Kaplan–Meier methods, and Cox regression analysis was applied to explore associations between clinical factors and survival. A total of 114 patients were analyzed. The median patient age was 51 years (range: 18–78 years), and 60.5% were male. Gross total resection was performed in 76 patients prior to RT. IDH1/2 wild-type was identified in 99 patients, and MGMT methylation was present in 40 patients. The median follow-up period was 34.0 months (95% CI, 27.1–40.9 months). The median overall survival (OS) and progression free survival (PFS) were 30.0 months (95%. CI 21.5–38.5 months), and 12.0 months (95%CI 9.5–14.5 months), respectively. Both univariate and multivariate analyses showed that the type of surgical resection and IDH1/2 mutation status were significantly associated with OS and PFS. Headache and fatigue were the most frequently reported symptoms. Delivering a RT boost to high-risk tumor regions identified by MRS after standard RT is feasible in patients with GBM and demonstrate acceptable toxicity. Further prospective clinical trials are warranted to confirm these findings.

## Introduction

Glioblastoma (GBM) is the most frequent primary malignant brain tumor in adults. The current standard of care for newly diagnosed GBM involves maximal surgical resection, followed by adjuvant radiotherapy (RT) with concurrent daily temozolomide, and six cycles of adjuvant temozolomide therapy^1^. The EORTC study reported a median survival of approximately 14.6 months^2^ for patients with GBM. Despite aggressive neurosurgical resections and RT, patients almost always experience recurrence near or within the tumor bed. A phase II study demonstrated that a stereotactic radiosurgery (SRS) boost in addition to conventional RT for high-grade gliomas was feasible and potentially effective. However, the Phase III trial (RTOG 93–05) failed to show a survival advantage from boosting the contrast-enhancing surgical bed^3^. The possible cause for this failure was the lack of precision in target volume delineation. As a results, the optimal approach to SRS boost therapy in GBM remains uncertain and warrants further evaluation.

Magnetic resonance spectroscopy (MRS) is based on nuclear magnetic resonance technique to investigate the metabolism of chemicals in the body. Proton magnetic resonance spectroscopy (1H MRS) has been widely used in the diagnosis of gliomas. In gliomas, *N*-acetyl aspartate (NAA) is typically reduced due to neuronal destruction caused by the tumor, while Choline (Cho) levels are elevated as a result of tumor cell proliferation. Consequently, an abnormal Cho/NAA ratio is often observed in areas of tumor infiltration. MRS can more accurately define the boundaries of high-grade gliomas compared to conventional MRI, as demonstrated by histopathologic correlation^4^. The use of MRS to define target volumes for RT treatment planning holds potential to improve control while reducing complications^5^. Recent efforts have focused on accurately identifying high-risk regions within high-grade gliomas that appear more aggressive and may benefit from an additional targeted limited-volume RT boost without the increased risk of toxicity associated with large-volume treatments. A phase II study showed that patients treated with MRS-guided boost RT had a median survival of 20.8 months, compared to the historical control of 14.6 months in patients receiving conventional RT and temozolomide. In this study, 35 GBM patients had MRS used to outline the tumor bed after surgery, with radiation doses increased to between 66 and 81 Gy to the gross tumor volume (GTV)^6^.

The purpose of this retrospective study was to determine the feasibility and efficacy of delivering a radiation dose boost to high-risk tumor regions as determined by MRS after standard RT for patients with GBM.

## Methods and materials

### Patient selection

This retrospective study included 114 patients newly diagnosed with GBM between January 2017 and July 2023. The study was approved by the institutional review board of Sichuan Cancer hospital, and all patients signed written informed consent. The study was performed in accordance with the Helsinki Declaration and its later amendments. Diagnosis of GBM was confirmed for all patients through either biopsy or surgical resection, according to the 2016 World Health Organization (WHO) classification of central nervous system (CNS) tumors. Patients had a Karnofsky Performance Status (KPS) score ≥ 60 and were treated according to the Stupp protocol^1^, which was followed as the standard therapeutic regimen.

## Treatment

**Fig. 1:**
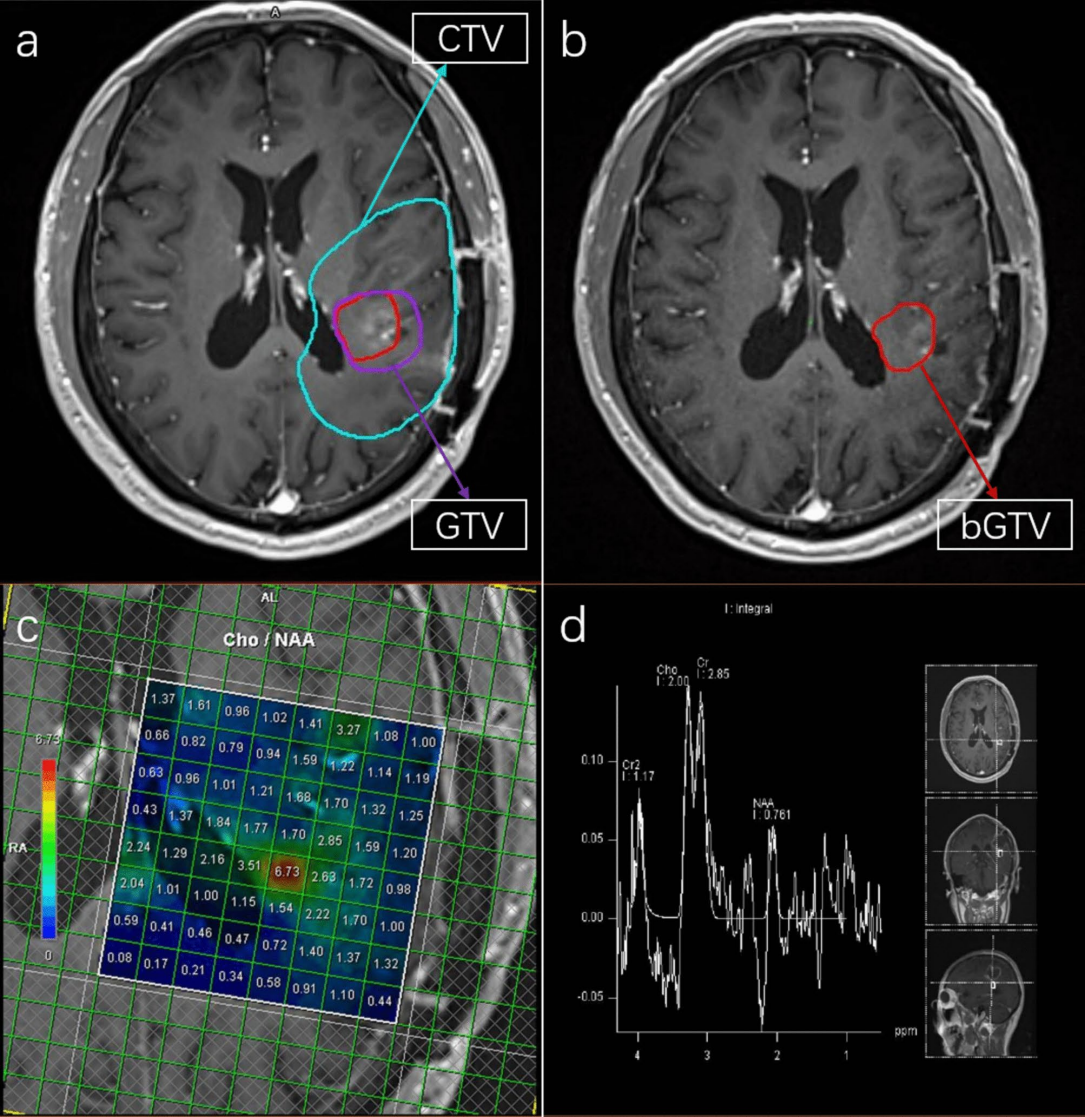
High-risk tumor regions as determined by MRS after standard radiotherapy in patients with GBM: (**a**) T1-weighted sequence before radiotherapy; (**b**) bGTV in T1-weighted sequence after a 60 Gy dose; (**c**, **d**) regions of interest showing the Cho/NAA ratio.

All patients underwent Intensity-modulated radiotherapy (IMRT) in combination with temozolomide. Radiotherapy was delivered with a prescription dose of 60Gy in fractions of 2.0Gy. All patients underwent MRI/MRS both prior to the start of radiotherapy and after the 60Gy dose. Planning target volumes were derived from dedicated CT or MRI scans of the whole brain. For the first 60Gy in 30 fractions according to ESTRO-ACROP guideline^7^, the treatment volume encompassed the contrast-enhancing lesions and surrounding edema as visualized on postoperative MRI scans (Fig. 1). Treatment plans included either intensity modulated radiation therapy (IMRT) or volumetric modulated arc therapy (VMAT). Critical structures were monitored to meet the dose constraints: brainstem to 60Gy, lenses to 10Gy, optic nerves to 55Gy, optic chiasm to 60Gy and cochlea to 55Gy.

## MRS image processing

All patients underwent brain MRS with a voxel size of 10 × 10 × 15 mm before radiotherapy and after receiving a total dose of 60Gy. All examinations were carried out on MRIs equipped with the CSI 3D module on a 3.0 T MRI scan. The acquisition parameters were as follows: TR/TE = 1520/135 ms, FOV = 120 mm × 120 mm × 100 mm, matrix size = 12 × 12 × 8, slice thickness = 40.0 mm, and a number of excitations (NEX) = 2. The voxel size was approximately 1.0 cm × 1.0 cm × 1.5 cm, consistent across both acquisition and reconstruction. The 1H-MRS scan time was approximately 8 min and 58 s. The entire area displaying T1/T2 abnormalities on postoperative scans was analyzed. Spectrographic peaks for choline, N-acetyl aspartate (NAA), and creatine were identified, with choline and NAA levels normalized to baseline creatine levels. Voxels exhibiting a normalized choline/NAA ratio greater than 2 were manually identified and marked (Fig. 1). These marked voxels were then highlighted on the T1-weighted sequence. Subsequently, a biological gross target volume (bGTV) was delineated on the simulation CT using the image fusion with the T1 weighted sequence and MRS data following the administration of 60Gy.

## Boost radiotherapy

The prescription dose for all patients ranged from 10 to 20Gy, according to the volume of bGTV (10 Gy for diameters 3–4 cm, 14 Gy for diameters 2–2.9 cm, and 20 Gy for diameters < 2 cm) Patients presenting with symptoms of increased intracranial pressure received an intravenous (i.v.) loading dose of dexamethasone and mannitol prior to radiotherapy.

## Patient follow-up and statistical analysis

Follow-up period was calculated from the completion of radiotherapy. All patients received brain MRI/MRS every 2–3 months after the completion of RT. Overall survival (OS) was measured from the start of RT treatment to the date of death, with survivors being censored at the date of their last follow-up. Progression-free survival (PFS) was defined as the time from the date of RT to either disease progression or death from any cause. The Kaplan–Meier method was used to estimate OS and PFS, with comparisons between groups made using the log-rank test. Toxicity was graded according to the National Cancer Institute Common Toxicity Criteria, version 5.0. All statistical analyses were performed using the SPSS version 25.0 (IBM SPSS Statistics, Chicago, IL).

## Results

### Patient characteristics

The detailed characteristics of the 114 patients included in the study are presented in Table 1. The median age of patients was 51 years (range 18–78), with 60.5% being male. The median KPS was 90. A total of 76 patients underwent gross total resection prior to RT. Among the cohorts, 99 patients had IDH1/2 wild-type status, and 40 patients presented MGMT promoter methylation. Half of the patients received a boost of 10Gy.

**Table 1:** Characteristics of patients.

Patients	N (%)
Number	114
Age, years	
Median (Range)	51 (18–78)
Gender	
Male	69 (60.5)
Female	45 (39.5)
KPS	
Median (Range)	90 (60–100)
Surgical resection	
Biopsy	7 (6.1)
Subtotal	31 (27.2)
Gross total	76 (66.7)
MGMT promoter methylation status	
Methylated	40 (35.1)
Unmethylated	49 (43.0)
Undetermined	25 (21.9)
IDH1/2 mutation status	
Mutant	15 (13.2)
Wild-type	99 (86.8)
Tumor Location	
Frontal	36 (31.6)
Temporal	35 (30.7)
Parietal	14 (12.3)
Occipital	9 (7.9)
Insular	17 (14.9)
Brainstem	3 (2.6)
Boost dose	
10Gy	57 (50.0)
14Gy	37 (32.5)
20Gy	20 (17.5)
The number of courses of maintenance TMZ therapy	
no	1 (0.9)
< 6	36 (31.6)
6–12	24 (21.1)
> 12	53 (46.5)

Abbreviations: KPS = Karnofsky performance status, MGMT = methylguanine-DNA methyltransferase, IDH = isocitrate dehydrogenase, TMZ = Temozolomide.

## Survival

**Fig. 2:**
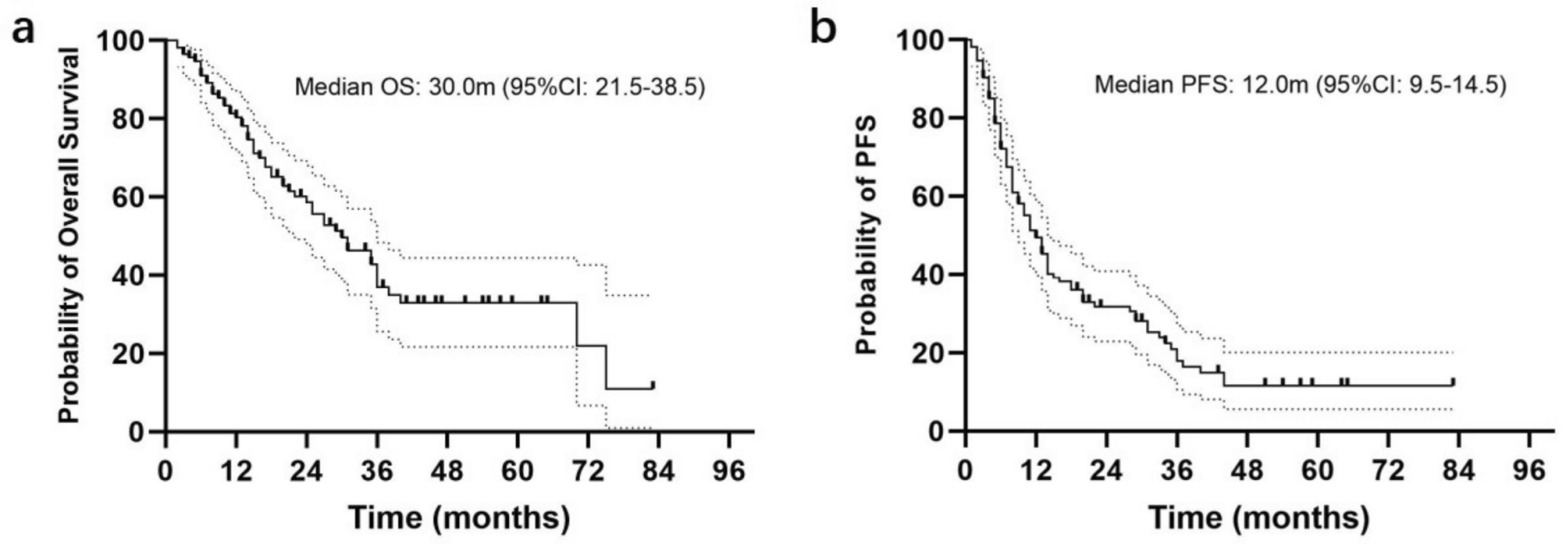
Kaplan–Meier curves for overall survival (**a**) and progression-free survival (**b**) for all patients.

**Fig. 3:**
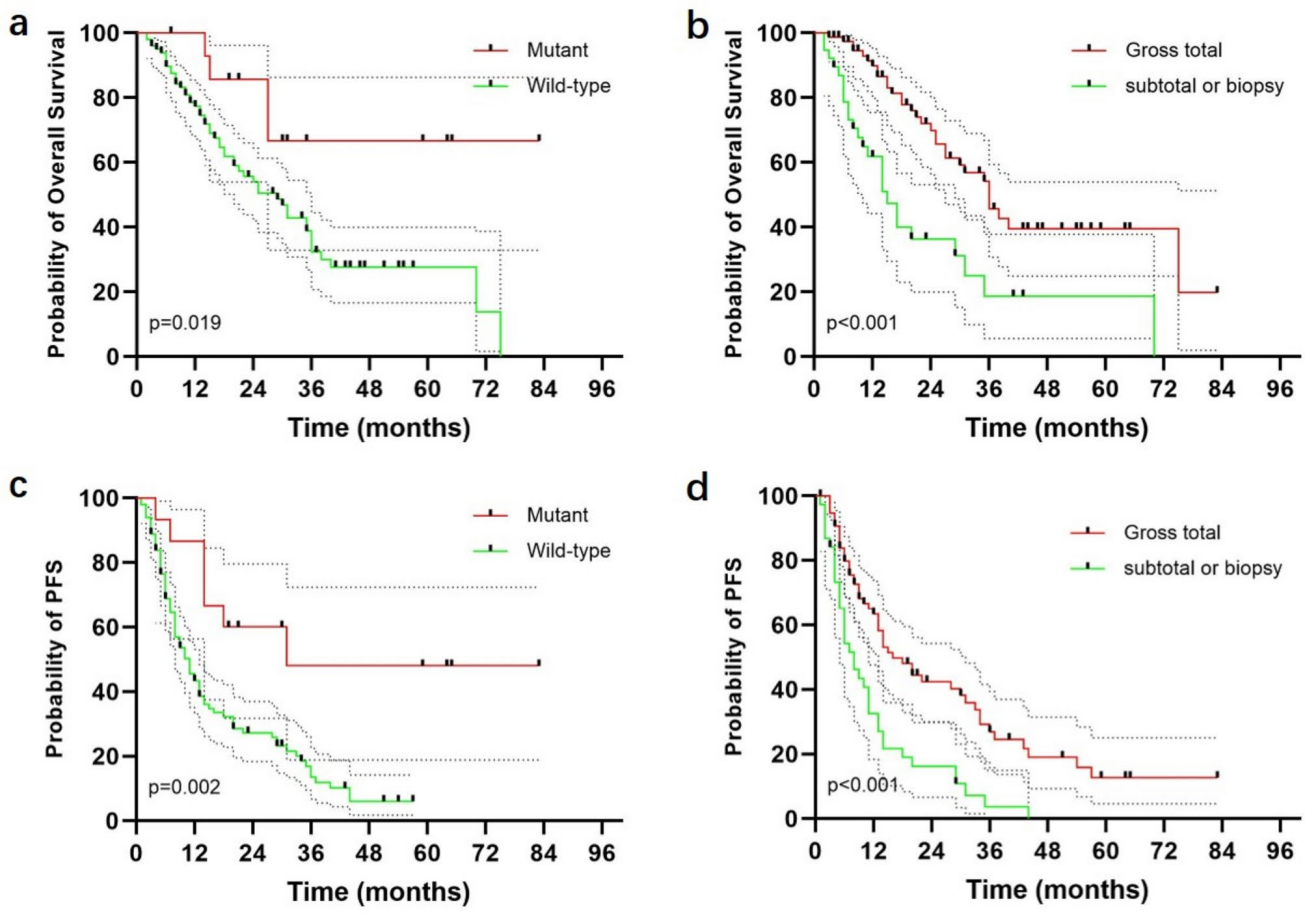
Overall survival and progression-free survival by IDH1/2 mutation status (**a**, **c**) and surgical resection type (**b**, **d**).

The median follow-up duration was 34.0 months (95% CI, 27.1–40.9 months). The median OS and PFS for the entire cohort were 30.0 months (95%CI 21.5–38.5 months) and 12.0 months (95%CI 9.5–14.5 months), respectively (Fig. 2). A significant association between OS, PFS, and both the type of surgical resection and IDH1/2 mutation status was observed. Patients who underwent gross total resection had a median OS of 36 months, compared to 15 months for those who underwent subtotal resection or biopsy (p < 0.001) (Fig. 3). Patients with IDH1/2 mutation had longer median OS and PFS compared to those with wild-type variant (p = 0.019, p = 0.002). No significant difference in median OS was detected between patients with and without MGMT promoter methylation (p = 0.795). Other factors did not show statistically significant. For IDH1/2 wild-type patients, the median OS and PFS were 29.0 months (95%CI 20.7–37.2 months) and 11.0 months (95%CI 8.0–14.0 months), respectively. The type of surgical resection was the only prognostic factor for OS and PFS (p = 0.001, p = 0.002).

## Treatment toxicity

Overall, the incidence of treatment-related adverse events (AEs) of any grade is showed in Table 2. Headache and fatigue were the predominant symptoms among all patients. A total of 25 patients experienced grade 3 or higher neurological toxicities, including severe headache and vomiting, which required hospitalization. Hematological adverse events did not exceed grade 3. No treatment-related deaths occurred.

**Table 2:** Treatment-related adverse events according to CTCAE 5.0

Toxicity (n = 114)	Grade 1	Grade 2	Grade 3	Grade 4
Headache	16	37	20	1
Fatigue	44	33	15	0
Vomiting	23	14	8	0
Anemia	44	21	0	0
Leukopenia	52	18	0	0
Thrombocytopenia	19	15	5	0

## Discussion

GBM is a highly aggressive brain tumor characterized by relatively poor OS. The median survival within the Stupp protocol was approximately 14.6 months in the EORTC study^1^. Although OS has improved since the introduction of tumor treating fields (TTFs) in the management of newly diagnosed GBM, the prognosis remains dismal, with 5-year OS rates ranging from 2 to 10%^8^. Several prospective, single-arm, dose-escalation trials have been conducted to improve survival outcomes^6,9–14^. A multi-institutional pilot clinical trial of MRS-guided radiation dose escalation for newly diagnosed glioblastoma enrolled 30 patients. The results showed that dose-escalated RT to 75 Gy guided by MRS appeared feasible and safe for the GBM patients^15^. A recent systematic review of 22 prospective trials, comprising a total of 2198 patients, found a survival benefit with RT dose escalation alone, but no significant improvement with higher dose combined TMZ ^16^.However, the NRG BN-001 prospective randomized trial showed no significant improvement in PFS or OS with IMRT dose-escalated compared to standard RT^17^.

Our retrospective study aimed to evaluate the efficacy of MRS targeted boost radiotherapy in the treatment of patients with GBM. The median survival in our cohort, using MRS targeted boost RT was 30.0 months, which represents a substantial improvement over expected survival based on historical controls^2^. Compared to another phase 3 clinical trial^18^ where patients receives a simultaneous integrated boost totaling 72Gy to MRSI metabolic abnormalities, the tumor bed and residual contrast enhancements, our cohort demonstrated superior median survival. These findings may be partly attributed to the higher percentage of patients (66.7%) who underwent gross total resection in our study. Surgical resection type was identified as significant prognostic factor in both univariate and multivariate analyses. In addition, our cohort received close clinical follow-up and frequently underwent one or more surgeries, often including treatments with bevacizumab and re-irradiation upon tumor recurrence.

In recent years, functional imaging techniques such as MRS and PETCT have been increasingly used in glioblastoma^12,19,20^. The use of a functional image-guided boost is more selective and specific for targeting tumor tissue rather than post-surgical changes, potentially resulting in improved efficacy compared to boosting areas based solely on contrast enhancement. In our study. metabolic alterations observed at the end of standard RT were used to guide the boost treatment, which may have allowed for more accurate detection of residual lesions. This approach also helps mitigate issues related to tumor position shifts during treatment, such as reduction of the postsurgical cavity in the management of edema after irradiation.

Our results demonstrated a median PFS of 12.0 months (95%CI 9.5–14.5 months), which is superior to the expected outcomes based on historical EORTC data. Additionally, the functional boost volume in our study was potentially smaller than those reported in previous studies^6,17–19^, possibly contributing to the favorable outcomes observed. Importantly, all patients were able to complete the boost RT, and no significant increase in toxicity or side effects was observed during follow-up.

Our study had several limitations. First, this was a single arm study without a control group, and the sample size was limited. In addition, the retrospective nature of the led to variability in the doses of boost RT, as they were not uniform across the cohort. Furthermore, the administration of boost RT after the end of standard radiotherapy increased the workload for both physicians and physicists, as well as the overall cost of patient treatment. Finally, the patient’s long-term toxicity cannot be completely and accurately analyzed.

## Conclusions

In conclusion, our findings show that boost RT targeting high-risk tumor regions, as determined by MRS after standard radiotherapy for patients with GBM, is feasible and associated with acceptable toxicity levels. The survival outcomes for patients treated with MRS targeted RT are superior to historical results observed with standard treatment protocols. However, further randomized controlled trials are needed to evaluate the efficacy of mage-guided RT in GBM patients.

## Data Availability

The datasets used and/or analysed during the current study available from the corresponding author on reasonable request.

## References

[1] Stupp, R. et al. Radiotherapy plus concomitant and adjuvant temozolomide for glioblastoma. *N. Engl. J. Med.* **352**, 987–996 (2005). 15758009 10.1056/NEJMoa043330

[2] Mirimanoff, R.-O. et al. Radiotherapy and temozolomide for newly diagnosed glioblastoma: Recursive partitioning analysis of the EORTC 26981/22981-NCIC CE3 Phase III randomized trial. *J. Clin. Oncol.* **24**, 2563–2569 (2006). 16735709 10.1200/JCO.2005.04.5963

[3] Souhami, L. et al. Randomized comparison of stereotactic radiosurgery followed by conventional radiotherapy with carmustine to conventional radiotherapy with carmustine for patients with glioblastoma multiforme: Report of radiation therapy oncology group 93–05 protocol. *Int. J. Radiat. Oncol.* **60**, 853–860 (2004). 10.1016/j.ijrobp.2004.04.011 15465203

[4] Croteau, D. et al. Correlation between magnetic resonance spectroscopy imaging and image-guided biopsies: Semiquantitative and qualitative histopathological analyses of patients with untreated glioma. *Neurosurgery.* **49**, 823–829 (2001). 11564242 10.1097/00006123-200110000-00008

[5] Pirzkall, A. et al. MR-spectroscopy guided target delineation for high-grade gliomas. *Int. J. Radiat. Oncol. Biol. Phys.* **50**, 915–928 (2001). 11429219 10.1016/s0360-3016(01)01548-6

[6] Einstein, D. B. et al. Phase II trial of radiosurgery to magnetic resonance spectroscopy-defined high-risk tumor volumes in patients with glioblastoma multiforme. *Int. J. Radiat. Oncol. Biol. Phys.* **84**, 668–674 (2012). 22445005 10.1016/j.ijrobp.2012.01.020 PMC4334318

[7] Niyazi, M. et al. ESTRO-ACROP guideline “target delineation of glioblastomas”. *Radiother. Oncol. J. Eur. Soc. Ther. Radiol. Oncol.* **118**, 35–42 (2016). 10.1016/j.radonc.2015.12.003 26777122

[8] Stupp, R. et al. Effect of tumor-treating fields plus maintenance temozolomide vs maintenance temozolomide alone on survival in patients with glioblastoma: A randomized clinical trial. *JAMA.* **318**, 2306–2316 (2017). 29260225 10.1001/jama.2017.18718 PMC5820703

[9] Mallick, S. et al. Hypo-fractionated accelerated radiotherapy with concurrent and maintenance temozolomide in newly diagnosed glioblastoma: Updated results from phase II HART-GBM trial. *J. Neurooncol.* **164**, 141–146 (2023). 37452916 10.1007/s11060-023-04391-7

[10] Scoccianti, S. et al. Hypofractionated radiotherapy with simultaneous integrated boost (SIB) plus temozolomide in good prognosis patients with glioblastoma: a multicenter phase II study by the Brain Study Group of the Italian Association of Radiation Oncology (AIRO). *Radiol. Med. (Torino)* **123**, 48–62 (2018). 28879459 10.1007/s11547-017-0806-y

[11] Tsien, C. I. et al. Concurrent temozolomide and dose-escalated intensity-modulated radiation therapy in newly diagnosed glioblastoma. *Clin. Cancer Res*. *Off. J. Am. Assoc. Cancer Res.* **18**, 273–279 (2012). 10.1158/1078-0432.CCR-11-2073 PMC3266840 22065084

[12] Piroth, M. D. et al. Integrated boost IMRT with FET-PET-adapted local dose escalation in glioblastomas. Results of a prospective phase II study. *Strahlenther. Onkol. Organ Dtsch. Rontgengesellschaft Al.* **188**, 334–339 (2012). 10.1007/s00066-011-0060-5 22349712

[13] Monjazeb, A. M. et al. A phase I dose escalation study of hypofractionated IMRT field-in-field boost for newly diagnosed glioblastoma multiforme. *Int. J. Radiat. Oncol. Biol. Phys.* **82**, 743–748 (2012). 21236604 10.1016/j.ijrobp.2010.10.018 PMC4586107

[14] Douglas, J. G. et al. [F-18]-fluorodeoxyglucose positron emission tomography for targeting radiation dose escalation for patients with glioblastoma multiforme: clinical outcomes and patterns of failure. *Int. J. Radiat. Oncol. Biol. Phys.* **64**, 886–891 (2006). 16242251 10.1016/j.ijrobp.2005.08.013

[15] Ramesh, K. et al. A multi-institutional pilot clinical trial of spectroscopic MRI-guided radiation dose escalation for newly diagnosed glioblastoma. *Neuro-Oncol. Adv.* **4**, vdac006 (2022). 10.1093/noajnl/vdac006 PMC8976280 35382436

[16] Singh, R. et al. Dose escalated radiation therapy for glioblastoma multiforme: An international systematic review and meta-analysis of 22 prospective trials. *Int. J. Radiat. Oncol. Biol. Phys.* **111**, 371–384 (2021). 33991621 10.1016/j.ijrobp.2021.05.001

[17] Gondi, V. et al. Radiotherapy (RT) dose-intensification (DI) using intensity-modulated RT (IMRT) versus Standard-dose (SD) RT with Temozolomide (TMZ) in newly diagnosed glioblastoma (GBM): Preliminary results of NRG oncology BN001. *Int. J. Radiat. Oncol.* **108**, S22–S23 (2020).

[18] Laprie, A. et al. Randomized phase III trial of metabolic imaging-guided dose escalation of radio-chemotherapy in patients with newly diagnosed glioblastoma (SPECTRO GLIO trial). *Neuro-Oncol.* **26**, 153 (2024). 37417948 10.1093/neuonc/noad119 PMC10768994

[19] Madan, R. et al. Prospective phase II study of radiotherapy dose escalation in grade 4 glioma using 68Ga-pentixafor PET scan. *Clin. Oncol. R. Coll. Radiol. G. B.* **36**, e294–e300 (2024). 10.1016/j.clon.2024.04.011 38821722

[20] Harat, M. et al. Safety and efficacy of irradiation boost based on 18F-FET-PET in patients with newly diagnosed glioblastoma. *Clin. Cancer Res Off. J. Am. Assoc. Cancer Res.* **28**, 3011–3020 (2022). 10.1158/1078-0432.CCR-22-0171 35552391

